# Meditations on Methodological Issues in Clinical Investigations of “Central Sensitization” (Jonah XII)

**DOI:** 10.3390/jcm15166174

**Published:** 2026-08-09

**Authors:** Shiloh Plaut

**Affiliations:** 1 Department of Basic and Clinical Sciences, University of Nicosia Medical School, Nicosia 2408, Cyprus; shilowhale@gmail.com; Tel.: +357-22-471900; 2 Meuhedet Health Services, Jerusalem 9434171, Israel

**Keywords:** pain mechanisms, pain research, central sensitization, research methodology, study design, measurement, fibromyalgia

## Abstract

This perspective paper addresses five methodological concerns in clinical investigations across the field of pain research and the paradigm of central sensitization. The first is the conflation of the phenomenon and the measurement tool: a patient reported outcome questionnaire called “Central Sensitization Inventory” is given as an example. Second, instruments that are used for measuring central sensitization (and its severity) are not validated to measure the phenomenon of central sensitization. The third is conceptual ambiguity regarding the theoretical framework of central sensitization and nociplastic pain, perpetuating the perception of central sensitization/nociplastic pain as both a phenomenon and a mechanism at the same time. The fourth is the conflation of clinical reasoning with scientific thinking, where in the former jumping from the mechanism to the phenomenon and vice versa is natural. The fifth is terminology issues such as “central-sensitivity syndromes” and “central sensitization-associated symptoms” when referring to fibromyalgia and similar conditions. In summary, by clinician-researchers confusing empirics with theory and theory with empirics and using clinical reasoning instead of scientific thinking, central sensitization becomes an axiom instead of a paradigm to be investigated according to hypothesis-driven studies. The failure to be aware of and clearly distinguish between these cognitive frameworks can lead to flawed research designs, inappropriate interpretations of findings, groundless mechanistic conclusions, and ultimately compromised clinical decision making.

## 1. Prologue

Dear editor,

This manuscript raises methodological concerns I encountered during my review of the medical literature on central sensitization (CS) across the field of neuroscientific pain research. Five issues are worth mentioning which I reckon might be relevant to some of your readership.

## 2. Problem #1: Conflating the Measurement Tool with the Phenomenon

The first issue pertains to empirical studies that inappropriately rely on the Central Sensitization Inventory tool as a research tool for investigating CS [1,2,3,4,5,6,7,8]. In empirical studies, the central sensitization inventory is used as a measurement tool for central sensitization. These empirical studies are designed with circular reasoning in their attempt to investigate central sensitization [3,9,10,11,12,13].

The Central Sensitization Inventory (CSI) [14,15], originally developed by Mayer and Neblett et al. (2011), is frequently used in research as a tool to assess symptoms associated with “central sensitivity syndromes” (or “central sensitization associated symptoms”) [16,17,18,19,20,21,22,23,24,25], such as fibromyalgia (FM), and recently applied to endometriosis [1], osteoarthritis [10], primary Sjögren’s syndrome [11], rheumatoid arthritis [13], spondyloarthritis [26], Raynaud’s phenomenon [27], inflammatory bowel disease [28], migraine [7], and long-covid syndrome [2]. This tool has been widely used by clinicians and researchers from a multitude of fields globally for the past ten years (reaching even obstetrics and gynecology, psychology, neurology, gastroenterology, orthopedics, dermatology, emergency medicine, nursing, physiotherapy, and more), including in papers published in leading peer-reviewed international academic medical journals [29,30,31,32,33]. It is regarded as a useful instrument to measure CS and its severity both by researchers in the field [1,3,7,8,31,34,35], and by experts and clinicians (e.g., Delphi study) [36,37]. However, its methodological foundation raises significant issues. The problem is that authors correlate constructs such as pain, anxiety, and depression— that of which are all found in the CSI questionnaire—with the CSI score and test for unavoidable correlations, mistakenly interpreting these as correlations with central sensitization.

The CSI is a symptom-based questionnaire that assesses patients’ self-reported clinical manifestations, mainly inquiring about stress, pain of several sorts (jaw pain, pelvic pain, widespread pain), fatigue, hypervigilance, disturbed sleep, and low mood/affect (with 25 items rated on a Likert scale from 0 to 4 with the same weight for each item), as well as the presence of other functional somatic syndromes. This questionnaire, however appears to lack a theoretical basis grounded in the underlying mechanisms of CS. Instead, the tool was constructed mainly based on knowledge of the symptoms experienced by patients with chronic pain and the expertise of healthcare personnel dealing with patients in the clinical setting. As described for its construction process by Mayer and Neblett et al. in their publication’s methods section: [14] *“An interdisciplinary team that included physicians (psychiatrists and orthopedic surgeons), rehabilitation specialists, clinical psychologists, health psychologists, and psychophysiological specialists, who work exclusively with individuals with chronic pain conditions, developed the items for this Inventory.”* While this method offers valuable insights into clinical manifestations and symptomatology, it lacks the methodological rigor typically expected from instruments used for measuring neurophysiological or pathobiological phenomena.

“Central sensitization” [38] refers to a neurological dysfunction of the central nervous system that exhibits abnormal hypersensitivity to sensory input and amplified response to noxious and non-noxious stimuli independent of peripheral nociceptor activation, due to structural and functional alterations in the spinal cord dorsal horn and brain, postulated to involve a combination of neuronal hyperexcitability, impaired descending inhibitory control, glial reactivity, and complex neuropathological processes of dysregulated central pain processing pathways [31,39,40,41,42,43,44]. Despite being the most accepted theory for the pain, hyperalgesia, and allodynia of “primary fibromyalgia syndrome”, the theory of CS, as it is currently theorized, does not explain the complex collection of symptoms and anomalies in fibromyalgia (or “functional somatic syndromes”) demonstrated in empirical research [45,46,47,48,49,50,51,52].

A valid instrument for measuring nociplasting central pain augmentation or centralization of pain must be mechanistically derived from, or at least conceptually anchored in, the above theoretical principles—ones postulated for what pain researchers seem to refer to as “nociplastic pain”. However, the CSI questionnaire does not measure such neurophysiological mechanisms. Instead, its items are based on established clinical diagnoses and symptom patterns that, although they may result from CS (and/or chronic pain and stress), are neither exclusive to it nor necessarily indicative of its causal pathways.

The theory of the pathophysiology of fibromyalgia is still, as far as I am aware, under dispute across the field [45,46,47,51,53,54,55].

This misalignment seems to have three significant implications:

1. Mechanistic misinterpretations and circular reasoning: First, the use of the CSI risks conflating symptom clusters and mechanistic insights, potentially leading to misinterpretations regarding the role of central sensitization both in scientific research and in clinical practice. Researchers and clinicians are erroneously using the CSI as a scientific measurement instrument to identify [2,7,26,29,34,56,57,58,59,60,61,62,63,64], assess [1,6,56,65,66], and measure [5,31,35,36,57,67,68,69] CS and its severity. Table 1 and Table 2 below list several examples. I allow myself to give an example of a study briefly, just to demonstrate the extent of the problem, particularly from the field of psychology, published in one of the top ranked journals in the field. Studies published in international leading journals in pain research also have similar methodological issues.
  A study [3] aimed “to test whether the associations between level of pain and CS were independent of or mediated by emotional distress” in a population of chronic pain patients via an online survey and included adults with pain for at least three months (i.e., chronic pain). Pain intensity was assessed using a composite pain index, emotional distress was assessed using a Spanish version of the Depression Anxiety Stress Scale, and CS severity was assessed using the Spanish version of the CSI (the inventory which consists of symptomatology of chronic pain/fibromyalgia-type patients, such as pain, sleep disturbances, fatigue, functional impairment, psychosomatic symptoms, and emotional distress). Basically, they set out to assess a group that contains participants with phenomenon X (chronic pain/fibromyalgia-type symptoms) and compared them against phenomenon Y (“central sensitization-associated symptoms”), without realizing that X and Y are represented by same constructs, and, unsurprisingly, found a significant correlation. This may indeed be an interesting finding to discuss, but that was not what the study was designed to do. They did not aim to study the impact of fibromyalgia-type manifestations. They wanted to study central sensitization, a disease of the central nervous system. They concluded that CS was identified in their population and that CS and emotional distress are independently associated with pain. I imagine they chose to use the CSI since they saw that the tool is being used across the medical fields to measure CS and, therefore, probably assumed that the tool has already been validated to measure the phenomenon (i.e., hyperexcitability of nociceptive neurons and central nervous system pain augmentation). Obviously, nobody is perfect.
  In a peer-reviewed publication in a leading pain journal (Liu et al., 2024) [1], the study’s aim was to correlate mental health outcomes during the COVID-19 pandemic with pain-related phenotyping for central nervous system sensitization in endometriosis-associated pain. The primary outcomes were depression (PHQ-9) and anxiety (GAD-7) scores during the pandemic. The explanatory variables of interest were the CSI score (0–100) and endometriosis-associated chronic pain comorbidities/psychological variables before the pandemic. Then, they conducted a regression analysis. Basically, they set out to explain dependent variable/phenomenon Y (mental health/GAD/PHQ-9 during the pandemic) with independent explanatory variable/phenomenon X (“central sensitization”), without acknowledging that X and Y are represented by overlapping constructs, and, unsurprisingly, found a significant association. Item number 3 of the CSI questionnaire is “I have anxiety attacks” rated on a Likert scale, scored from 0 to 4. Item 16 of the CSI is “I feel sad or depressed.” Item 25 is “I have pain in my pelvic area” [70]. After multiple regression analysis, they found that increasing the CSI score by 10 was associated with an increase in pandemic PHQ-9 by 0.74 points (*p* < 0.0001) and GAD-7 by 0.73 points (*p* < 0.0001) on average. Consequently, they state that “endometriosis patients with a history of central sensitization before the pandemic had worse mental health outcomes during the COVID-19 pandemic” and “these associations remained significant in adjusted analyses” [1]. You can easily see that it is a fallacy because you have symptoms on one side of the equation and the same symptoms on the other side of the equation. That means you have a problem. Depression explains depression. Pelvic pain significantly correlates with pelvic pain. Chronic pelvic pain explains chronic pelvic pain. They conflate the empirics and the theory. The mechanism is not determined by terminology! High correlations are mathematically inevitable due to construct overlap, rendering the association biologically non-informative. The null hypothesis was never truly tested, yet the conclusion is central sensitization, irrespective of the actual results.
2. Terminology issues of CSI: The second misalignment with regards to the CSI concerns the terminology, which is misleading. The tool might more accurately be named the “Psychosomatic Pain and Stress Inventory”, “Fibromyalgianess Big Balagan symptomatology questionnaire”, or the “Fibromyalgic clinical index”—in coherence with its content and methodologic origin. There are many fibromyalgia questionnaires available in the literature [71,72,73,74,75,76]. Does only this one measure central sensitization?

Table 1 gives examples of studies using the CSI or relying on it inappropriately as a scientific tool for research of neuropathological central nervous system sensitization.

**Table 1:** Examples of studies using the CSI or relying on it as a tool in research on CS.

Publication Year, Journal, and Citation	Discipline/Department of Authors	Country	Citation (Section in Article)
2021, Lancet Rheumatology [31]	Anesthesiology and Pain Medicine, Orthopedic Surgery, Physical Medicine and Rehabilitation, Clinical Neuroscience, Physiotherapy	USA and multinational	“Patient-reported outcomes, such as the Central Sensitization Inventory (CSI), have also been used to measure central sensitisation, and they are practical to use in nearly every clinical setting….” (Diagnosis of central sensitisation) “In one study, preoperative central sensitisation persisted 2 years after total knee arthroplasty (n = 222), and patients showing preoperative central sensitisation (i.e., CSI score ≥ 40 [scale 0–100]; 55 of 222 patients) had worse quality of life, worse functional disability…”
2021, Journal of Neurology [56]	Department of Neurology, Department of Public Health	Japan	“… we hypothesized that CS could be associated with restless legs syndrome comorbidity in migraine patients.” (introduction) “Individuals with CSI-A scores ≥ 40 were defined as having CS.” (methods) “Patients with migraine showed a higher CSI Part A (CSI-A) score (30.0 ± 13.7 vs. 18.7 ± 12.4) and a higher rate of CS (21.0% vs. 8.6%) than healthy controls…” (results) “We showed that patients with migraine were 3 times more likely to have CS than healthy controls.” (conclusions)
2024, Best Practice and Research Clinical Rheumatology [59]	Rheumatology, Department of Biomedical and Clinical Sciences, Department of Medical Biotechnology and Translational Medicine	Italy	“In 2012 the Central Sensitization Inventory (CSI) was developed for the diagnosis of CS.” (Introduction Section 1.1. How to differentiate the type of pain in inflammatory rheumatic diseases)
2023, Pain Management Nursing [77]	Department of Physiotherapy	Spain	“Central sensitization was measured with the Central Sensitization Inventory (CSI).” (methods)
2024, RMD Open Rheumatic and Musculoskeletal Disorders [66]	Department of Rheumatology and Clinical Immunology, Department of Anesthesiology, Pain Center, Physiotherapy, and Rehabilitation Medicine	The Netherlands	“The Central Sensitisation Inventory (CSI) is a questionnaire developed and validated for the identification of CS.”, “Research in chronic pain conditions showed that a cut-off of ≥40 (range 0–100) is indicative of the presence of CS.” (introduction)
2022, Diagnostics [60]	Department of Orthopedic Surgery	South Korea	“The Central Sensitization Inventory (CSI), whole-body pain diagram, and quantitative sensory testing (QST) were used for measuring central sensitization.” (results Section 3.2)
2025, Journal of Patient-Reported Outcomes [78]	Department of Epidemiology and Biostatistics, School of Physical Therapy, Department of Surgery	Canada	“Although not all properties have been studied, the CSI, which received the highest overall ratings, could serve as a reliable patient reported outcome measure assessing CS in chronic pain” (abstract: conclusions)
2022, Best Practice and Research Clinical Rheumatology [79]	Department of Rheumatic and Immunologic Disease	USA	“A questionnaire-based study designed to explore the evidence of central sensitization as a potential mechanism for pain in a cohort of patients identified as having Long COVID revealed that 70.2% displayed evidence of such.” (Section heading: Pain)
2022, Pain Practice	Rheumatology Unit	Italy	“The CSI was used in the assessment of CS and pain sensitivity.” (Materials and Methods: Measurements)
2023, BMC Women’s Health [80]	Public Health, Centre for Translational Pain Research	Ireland	“In summary, 87% (n = 12) of participants had central sensitisation (CS) as per the Central Sensitisation Index with 57% (n = 8) above the cut-off score of >40 (more severe CS).” (abstract)
2023, The American Journal of Emergency Medicine [57]	Department of Emergency Medicine	USA	“The CSI is a validated survey consisting of two parts that can be used to accurately and efficiently determine whether a patient meets the criteria to be diagnosed with CS”, “At this point, the patient was given the Central Sensitization Inventory, and his score demonstrated severe underlying central sensitization.”
2021, JAMA Dermatology [29]	Department of Dermatology	The Netherlands	“Using a cutoff value 40 or higher as indication for the presence of CS has shown good test-retest validity with a sensitivity and specificity of 81% and 75%, respectively. The Dutch version of the CSI was validated with an intraclass correlation coefficient of 0.88 for patients with chronic pain and 0.91 for controls” (methods)
2021, Journal of Clinical Medicine [2]	Department of Neurosurgery, Center for Neurosciences, Faculty of Physical Education and Physiotherapy, Department of Physical Medicine and Rehabilitation, Department of Radiology	Belgium	“The goal of this study was to gain further insight in the presence of central sensitisation as underlying factor for long-term secondary effects in post COVID-19 patients.” (introduction) “Symptoms of central sensitisation were assessed with the Central Sensitization Inventory (CSI).” (methods) “This survey indicated the presence of symptoms of central sensitisation in more than 70% of patients post COVID-19 infection, suggesting towards the need for patient education and multimodal rehabilitation, to target nociplastic pain.” (abstract)
2016, Clinical Journal of Pain [67]	Department of Rehabilitation Sciences and Physiotherapy, Department of Clinical Neuropsychology, Transdisciplinary Pain Management Center	Belgium, The Netherlands	“The development of the CSI has clearly added substantially to the assessment of CS in clinical practice. The initial validation studies supporting the clinimetric properties of the CSI to measure CS underscore this notion” (introduction)
2023, Journal of Orthopedic Surgery and Research [69]	Department of Orthopedic Surgery	Japan	“The Central Sensitization Inventory (CSI) part A, which is a patient-reported outcome, was used to measure preoperative central sensitization.”
2024, Clinical and Experimental Rheumatology	Rheumatology	Italy	“Scores exceeding 60 on the Central Sensitization Inventory (indicative of extreme centralisation) were identified as…” [81]
2025, Pain Practice [82]	International Spine, Pain and Performance Center, School of Medicine and Health Sciences, College of Osteopathic Medicine	USA	“Evidence for central sensitization as classified by the central sensitization inventory in patients with pain and hypermobility” (main title)
2022, Pain [34]	Obstetrics and Gynecology	Canada	“The Central Sensitization Inventory (CSI) is a questionnaire previously validated in the chronic pain population…”, “In conclusion, a CSI ≥ 40 may be a practical tool to help identify patients with endometriosis with pain contributors related to central nervous system sensitization.” (Abstract)
2023, PLoS One [19]	Department of Rheumatology and Immunology, Department of Psychology	China	“The 9-item Central Sensitization Inventory (CSI-9) is a shortened version of the CSI-25, which is a patient-reported instrument used to screen people at risk of central sensitization (CS).” (abstract)
2021, Therapeutic Advances in Chronic Disease [64]	Physical Therapy, Nursing, Department of Health Sciences	Spain	“Central sensitization: The characteristic symptoms of patients with central sensitization syndrome (CSS) in conjunction with fibromyalgia syndrome were assessed using the Spanish version of the CSI.” (methods) “On the other hand, the mean and standard deviation of VAS, central sensitization, sleep quality, fatigue and anxiety were significantly higher in fibromyalgia syndrome women than in the controls (*p* < 0.001).” (results)
2019, Journal of Psychosomatic Research [83]	Department of General Practice and Elderly Care Medicine, Department of Primary and Community Care, Pain Management Center	The Netherlands	“Seven publications addressed the Central Sensitization Inventory (CSI), a 40 item self-report questionnaire that has been validated in several studies and that can be used both as a screener and as treatment outcome measure. Scerbo concluded in a systematic review in 2017 that the CSI generates reliable and valid data.” (results)
2023, BMC Musculoskeletal Disorders [6]	Rheumatology Department, Laboratory of Life and Health Sciences	Morocco	“The CSI score significantly correlated with self-reported function in knee osteoarthritis (KOA) patients and with physical performance in chronic low back pain (CLBP) patients. Several studies previously reported the same associations. A possible explanation for the correlation of CS with disability in KOA and CLBP might be the “fear avoidance model.” According to this theory, the development of chronic pain and disability is influenced by both neurophysiological processes related to pain sensitization and psychosocial factors such as pain catastrophizing.” (discussion)
2021, Lancet [84]	Rheumatology, Psychiatry, Behavioral Sciences, Anesthesiology and Critical Care Medicine, Neurology and Physical Medicine and Rehabilitation, Internal Medicine, Departments of Anesthesiology, Medicine, and Psychiatry, Chronic Pain and Fatigue Research Center, Department of Psychosomatic Medicine and Psychotherapy	Multinational	“The Central Sensitisation Inventory was designed to assess central sensitisation and can quantify symptom severity.” (Clinical evaluation)
2024, Turkish Journal of Physical Medicine and Rehabilitation [68]	Department of Physical Medicine and Rehabilitation	Turkey	“Neuropathic pain was assessed by the Self-Report Leeds Assessment of Neuropathic Symptoms and Signs (S-LANSS) scale and central sensitization was measured using the Central Sensitization Inventory (CSI)” (methods) “A total of 66.13% of the patients were diagnosed with nociceptive, 11.67% with neuropathic, and 22.20% with central sensitization.” (results)
2024, J Bodyw Mov Ther [85]	Physiotherapy, Neurology	Iran	“CS was evaluated using the Central Sensitization Inventory (CSI)” (abstract: methods). “Table 2 shows the intra- and inter-group change scores with 95% confidence intervals in terms of CS pre and post-intervention. CS scores were significantly reduced…” (results). “Using manual therapy in the clinical management of patients with migraine and neck pain may reduce the excitability of the CNS through inhibitory mechanisms which may help normalizing CS.” (discussion) “Based on our findings, there is preliminary evidence that migraineurs with neck pain have reduced level of CS following 4 sessions of upper cervical MT together with medication.” (conclusion)
2023, Clinical Journal of Pain [86]	Pain Center, Department of Anesthesia and Intensive Care, Nervous and Therapeutic Excitability research unit, Clinical Investigation Center, Institute of neurosciences	France	“Central sensitization, which was extreme in 69% of the patients (CSI score ≥ 60), was…”
2020, Clinical Rheumatology [58]	Department of Physical Medicine and Rehabilitation	Turkey	“We used the CSI to explore the prevalence of CS pain in patients with various rheumatic diseases and compared the results with those of FMS patients. CS was detected in nearly half of all patients with three different rheumatic diseases.” (discussion) “The CSI usefully detects CS pain in patients with rheumatic diseases.” (conclusions)
2020, RMD Open [87]	Pain Department, Rheumatology, Pain Evaluation and Treatment, Department of Physical Medicine and Rehabilitation	France	“The Central Sensitisation Inventory is a questionnaire that has been developed to identify the presence of pain of the central sensitisation type, regardless of a specific aetiology, with good psychometric characteristics.” (How to assess the presence of nociplastic pain)
2016, Journal of Pain [7]	Neuroscience Research, Brain and Mind Centre, and Department of Physical Faculty of Health Sciences	Australia	“Scores of ≥40 indicate possible central sensitivity syndromes, with higher CSI scores reflecting a higher degree of sensitization” (methods) “Of the variables having fair association with GABA levels, only CSI scores were suitable for ROC analysis because the migraine and the control groups had scores > 0 on the CSI.” (results) “Increased GABA levels were associated with higher central sensitization scores.” (discussion)
2024, Heliyon [30]	Department of Physical Therapy	Iran	“CSI is a self-reported tool to assess symptoms of CS” (methods). “This study aimed to determine the effect of dry needling on pain reduction, CS changes and psychological characteristics in women with CPP within a randomized clinical trial design…”, “The decrease of both CSI score and pain in DNG and PNG compared to the CG indicate that dry needling and placebo needling can change the sensitized central nervous system.” (discussion)
2021, BMC Primary care [36]	Department of General Practice	The Netherlands	“In a Delphi study among an international panel of experts, three tests for measuring CS were considered to be suitable for use in general practice: the Central Sensitization Inventory (CSI), pressure pain thresholds (PPTs) and monofilaments.” (Abstract: conclusions) “…some of the panellists expressed doubts about the construct validity of the test. The items of the CSI measure different constructs, like physical, psychological and cognitive functioning, physical symptoms and others. These constructs are probably related to CS, but further research is needed to establish the validity of the CSI” (discussion)
2024, Preprints database [88]	Department of Physical Therapy	Republic of Korea	“The central sensitization inventory (CSI) is a self-reported questionnaire developed and validated to assess patients with CS. In this study, the Korean version of the CSI (CSI-K) was used to evaluate the degree of CS among participants.” “Significant correlations between CSI-K and respiratory variables, pain variables, Muscle Tone, and Muscle Stiffness were observed between CSI-K and SEBQ (r = 0.719, *p* < 0.05), CSI-K and K-PCS (*p* < 0.05), and CSI-K and Muscle Stiffness (r = 0.546, *p* < 0.05)” “Multiple regression analysis for Muscle Stiffness with CSI-K, K-PCS, and Muscle Tone demonstrated statistical significance (F = 195.627, *p* < 0.05), with notably high explained variance of 97.8% (R^2^ = 0.978, adj R^2^ = 0.973). All three variables showed significant positive influences: CSI-K (β = 0.124, *p* < 0.05), K-PCS (β = 0.132, *p* < 0.05), and Muscle Tone (β = 0.861, *p* < 0.05)”
2024, Journal of Oral & Facial Pain and Headache [89]	Department of Experimental Dentistry, Department of Neurology, Statistical Analysis Center, Department of Stomatology and Maxillofacial Surgery, Department of Maxillofacial Orthopedics and Orthodontics	Poland, Slovakia	“The Central Sensitization Inventory (CSI) and the Somatic Symptom Scale-8 (SSS-8) were utilized to assess CS and the burden of somatic symptoms, respectively. (…) Higher levels of somatization were related to higher levels of CS and greater masticatory muscle pain.” (abstract)
2023, Annals of the Rheumatic Diseases [32]	Anesthesiology, Rheumatology, Immunology	USA	“Of particular relevance to the connection between Long COVID and FM is shared evidence of CNS dysfunction including glial activation and central sensitisation (Goudman et al., 2021) [2]…” (Pathophysiological links between FM and Long Covid, p. 137)
2024, Journal of Psychosomatic Research [90]	General and Internal Medicine, Psychology, Psychiatry, Social work	USA	“In a recent study, up to 70% of Long COVID patients had symptoms of Central Sensitization.” (introduction) “We developed a treatment program for Long COVID that targeted Central Sensitization utilizing CBT strategies to decrease pain and fatigue, improve sleep difficulties, improve cognitive functioning, and improve psychological distress” (introduction) “The program is a 16-h group-based multi-component class that targets the symptoms of Central Sensitization experienced by patients with Long COVID…” (methods) “The current paper describes an innovative multicomponent treatment program utilizing Cognitive Behavioral strategies to target symptoms of Central Sensitization in treating Long COVID.” (discussion) “There is mounting evidence supporting Central Sensitization as a causal or explanatory mechanism in developing Long COVID, much like other post-viral syndromes…” (discussion)
2024, Physiotherapy Research International [91]	School of Health Sciences	Portugal	“Also, our results do not support the assumption that having a more neuropathic component or increased central sensitization symptoms is associated with higher pain sensitivity or intensity.” (discussion)
2024, Reproductive sciences [92]	Gynecology Unit, Obstetric and Gynecological Emergency Unit, Department of Clinical Sciences and Community Health	Italy	“A PubMed and EMBASE systematic literature search… The Central Sensitization Inventory (CSI) is the most frequently used questionnaire for the detection of CS in patients with endometriosis. It has been validated in patients with endometriosis, in whom it appears to have good psychometric proprieties.” (abstract) “In fact, some authors argue these questionnaires are measuring psychological vulnerability and a hypervigilant state that is associated with pain, rather than CS itself.” (abstract)
2021, Pain [63]	Centre of Clinical Research Excellence in Spinal Pain, Injury and Health, Interdisciplinary Center for Research in Rehabilitation and Social Integration	Australia, Canada,	“The Central Sensitization Inventory (CSI) was developed to identify nociplastic pain and a cut-off score ≥ 40 is suggested to differentiate nociplastic from nociceptive pain.” (results: pain-type questionnaires)
2024, Journal of Rheumatology [93]	Rheumatology Clinic	Italy	“The CSI showed good clinimetric properties in different populations with chronic pain, and a cut-off of 40 points allows for correct identification in approximately 8 out of 10 patients with CS. The results reveal that a significant proportion of patients with PsA have symptoms compatible with CS regardless of the presence of coexisting FM…”
2024, BMC Primary Care [94]	Department of General Practice, Public Health research institute	The Netherlands	“GPs preferred the CSI because its results confirmed CS-related symptoms more frequently than those from the algometer and monofilament.” (abstract) “The CSI assisted GPs in initiating conversations about the symptoms associated with CS and how CS is explained. Patients reported feeling more understood as they recognized their symptoms reflected in the CSI’s questions.” (results)
2026, JAMA Network Open [95]	Neurology	USA	“Objectives: To analyze the prevalence of central sensitization syndrome (CSS) in postural tachycardia syndrome (POTS).” “Main outcomes and measures: Central Sensitization Inventory (to assess central sensitization syndrome.” “This study included 305 patients with POTS, of whom 264 (86.6%) met criteria for CSS” (Results) “These findings suggest that CSS was common in patients with POTS… This heightened central processing may amplify symptom perception through altered interoceptive signaling. Central sensitization and autonomic impairment may coexist, and management should focus on both conditions.” (conclusions)
2026, Joint Bone Spine [96]	Rheumatology	Italy	“The decrease in CSI-9 scores may reflect reduced central sensitization, potentially via descending pain inhibition mechanisms originating from vagus-activated nuclei.”

Table 2 shows the circular reasoning in empirical studies using the CSI inappropriately.

**Table 2:** Examples of empirical studies using the CSI inappropriately.

Publication Year, Journal, and Citation	Discipline/Department of Authors	Country	Citation (Section in Article)
2020, BMC Musculoskeletal Disorders [10]	Department of Orthopedic Surgery, Department of Rehabilitation	Japan	“…Thus, we aimed to determine the prevalence and characteristics of CS in patients with hip osteoarthritis (OA), in this study.” (Abstract: background) “The CS Inventory (CSI), used as a non-invasive routine clinical tool to evaluate the presence of CS 1 month before surgery in 100 patients with hip OA, was measured at our outpatient clinic, and the data were retrospectively reviewed.” (Abstract: methods) “The mean CSI score was 19.5 ± 11.3 and 5 (5.0%) of the patients had a score of 40 or more points. CSI scores correlated significantly only with VAS pain at rest (r = 0.348, *p* < 0.001).” (Abstract: results) “In this study, 1 out of every 20 hip OA patients had CS components.” (Abstract: conclusions)
2020, Journal of Arthroplasty [9]	Department of Orthopedic Surgery	South Korea	“Central sensitization (CS) has been recently identified as a significant risk factor for persistent pain and patient dissatisfaction following total knee arthroplasty (TKA). However, it remains unclear as to whether the preoperative CS persists after the elimination of a nociceptive pain source by TKA, or how CS affects the quality of life after TKA.” (Abstract: background) “All patients were preoperatively screened for CS using the Central Sensitization Inventory (CSI) and categorized into either a CS (n = 55; CSI ≥ 40) or non-CS group (n = 167; CSI < 40).” (Abstract: methods) “Two years after TKA, preoperative CS remained unchanged” (Abstract: results) “Preoperative CS was persistent at 2 years after TKA. Although CS patients achieved comparable clinical improvement following TKA, CS patients had worse quality of life, functional disability, and dissatisfaction than non-CS patients.” (Abstract: conclusion)
2025 Clinical Rheumatology [11]	Department of Physical Medicine and Rehabilitation, Division of Rheumatology	Turkey	“There is still a lack of understanding regarding the impact of central sensitization on the interpretation of disease activity and functional disability in primary Sjögren’s syndrome (pSS).” (abstract: background) “The Central Sensitization Inventory (CSI) was used to screen for central sensitization.” (abstract: methods) “The frequency of central sensitization was 65% in patients with pSS (n = 60).” (abstract: findings) “This study confirms that central sensitization has a major impact on functionality and the interpretation of self-reported disease activity in pSS.” (abstract: conclusions)
2023, Clinical Journal of Pain [86]	Pain Center, Department of Anesthesia and Intensive Care, Clinical Investigation Center, Institute of Neurosciences, Department of Physiology	France	“Central Sensitization and Small-fiber Neuropathy Are Associated in Patients With Fibromyalgia” (main title) “The objectives of this study were to determine the prevalence of electrochemical skin conductance abnormalities and to assess the CS in a larger cohort of patients with FM” (introduction) “According to the definition of “clinically relevant severity levels” proposed by Neblett et al., a CSI score ≥ 40 indicates a moderate CS (recommended to classify chronic pain patients as having CS) and a CSI score ≥ 60 indicates an extreme CS… Pearson correlation coefficients were used to assess the relationship between hESC/fESC…” (methods) “Regarding the CSI, 97.5% of the patients had clinically relevant central sensitization (CSI ≥ 40) and 69% of the patients had extreme central sensitization (CSI ≥ 60).” (findings) “According to the cutoff CSI score of 60, extreme CS was associated with the number of medications used (*p* = 0.020), antidepressant drugs (*p* = 0.021), and higher pain intensity (*p* < 0.001), FIQ (*p* < 0.001), NPSI (*p* < 0.001), or HADS (*p* < 0.001) scores” (findings) “This high level of CS likely explains the association of other determinants with ESC reduction, such as a higher HADS-depression score and medication intake, especially antidepressants, which may be a confounding factor in this case.” (discussion) “In conclusion, this study showed a significant association between the process of CS and the severity of small nerve fiber impairment in patients with FM.” (discussion)
2021, JAMA Dermatology [29]	Department of Dermatology	The Netherlands	“Main outcomes and measures: Based on current literature, a score of 40 or higher was deemed to indicate the presence of CS.” (abstract) “Conclusions and relevance: This new insight in the presence of CS in patients with hidradenitis suppurativa raises the question of whether we are adequately measuring and treating HS-associated pain.” (abstract)
2022, Seminars of Arthritis and Rheumatism [26]	Department of Rheumatology and Clinical Immunology	The Netherlands	“Disease activity and disease outcome were compared between groups with CSI score of <40 and ≥40 using Chi-square test, Independent Samples *t*-test, or Mann-Whitney U test for categorical, continuous normally distributed, and continuous non-normally distributed data, respectively.” (Methods) “The CSI, including the cutoff score of ≥40, is developed and validated to recognize chronic (nociplastic) pain in patients suffering from chronic pain and has already been used, but not validated, in osteoarthritis, RA, SpA, and also in IBD.”, “In conclusion, our cross-sectional study in long-term axSpA patients treated according to international standards in daily clinical practice demonstrated that CSI score indicative of CS has a major impact on disease-related QoL, also after correcting for potential confounders including disease activity. Awareness and treatment of CS in patients with axSpA has the potential to improve health-related QoL in these patients.” (Discussion)
2023, Journal of Pain research [97]	Department of Rehabilitation Medicine, Department of Orthopedics and Traumatology, Biomedical Innovation Center	China	“Central sensitization (CS) is commonly seen in chronic pain disorders, including neuropathic pain. However, there exist inconsistencies concerning the presence of CS in chronic pain secondary to carpal tunnel syndrome… this study aims to investigate the CS and pain profiles in patients with CTS…” (abstract: purpose) “The central sensitization inventory (CSI) was used to screen CS.” (abstract: patients and methods) “Over 60% of participants with CTS were found with clinical CS.” (results) “CS is prevalent in patients with CTS.” (conclusions)
2020, Pain Medicine [27]	Department of Physical Therapy, Department of Nursing, Rheumatology Division, Faculty of Health Science,	Spain	“Objective: To evaluate pain intensity, widespread pressure pain, central sensitization (CS), and catastrophizing between subjects with primary and secondary Raynaud’s phenomenon (RP) and healthy controls…” (objective) “Central Sensitization Inventory…” (methods) “Post hoc analysis revealed that secondary RP participants had a significantly higher level of CS compared with healthy controls (*p* = 0.001) and participants with primary RP (*p* = 0.001).” (results)
2018, Knee Surgery, Sports Traumatology, Arthroscopy [98]	Department of Orthopedic Surgery	South Korea	“Central sensitization is an abnormal enhancement of pain mechanism involving the central nervous system. Although psychological disorder is widely considered as a risk factor, the relationship between central sensitization and wound complication is currently unclear. Therefore, the purpose of this study was to investigate whether central sensitization was associated with high wound complication rate after primary total knee arthroplasty (TKA).” (purpose) “161 patients undergoing unilateral TKA were prospectively divided into two groups based on central sensitization inventory score of 40 points.” (materials and methods) “Multivariate logistic regression analysis showed that the odds of postoperative wound complications were increased 15.7 times in patients with central sensitization inventory score ≥ 40 (95% CI 4.1–60.0, *p* < 0.001).” (results) “Central sensitization is a risk factor for the development of postoperative wound complication after primary TKA.” (conclusions)
2024, EULAR meeting abstract, Annals of the Rheumatic Diseases [13]	Internal Medicine	Ukraine	“Pain is one of the most prominent symptoms of rheumatoid arthritis (RA), which significantly affects the functional capabilities of such patients and significantly worsens their quality of life. Recent evidence has demonstrated that Central Sensitization (CS) may be a new mechanism for the pain experienced by these patients.” (background) “Our aim was to determine the prevalence of central sensitization in RA patients using CSI.” (objectives) “We administered to all the subjects in the study the CSI, a questionnaire that has been used for the diagnosis of central sensitivity syndromes (CSS). DAS28-ESR, CDAI, SDAI, pain VAS, and HAQ-DI were compared in the “CS group (CSI ≧ 40)” and the “non-CS group (CSI < 40)”.” (methods) “ Overall, 176 patients with RA (median age, 52.2 ± 1.5 years; 78% female) and 36 controls (median age, 44.5 ± 2 years; 72% female) were included, of which 36% and 11%, respectively, had a CS (CSI score of 40 or higher), *p* < 0.001.” (results) “Central sensitization is common in RA patients.” (conclusions)
2024, EULAR meeting abstract, Annals of the Rheumatic Diseases [99]	Internal Medicine	Ukraine	“Axial spondyloarthritis (axSpA) is a chronic rheumatic disease characterized by intensive chronic pain syndrome, that can include different components—nociceptive, neuropathic and nociplastic. From the literature data, it is known that in 15% to 40% of patients with rheumatic diseases, the phenomenon of central sensitization (CS) may occur, which may be one of the mechanisms of nociplastic pain” (Background) “Objectives Our study aimed to determine the prevalence of CS in patients with axSpA and its influence on QoL and calculate the potential risk of FM. “To assess the intensity of pain, we used the VAS-Pain scale. To determine CS, the Central Sensitization Inventory (CSI) was used. The study was conducted in compliance with bioethical standards. All data were analyzed using IBM Statistics SPSS 23 software.” (Methods) “ Among 72 patients with axSpA, there were 21 persons (29.2%) with CS (CSI ≥ 40) with mean scores of CSI (M ± SD)—50.09 ± 11.4. Also, we calculated the potential risk of FM in axSpA patients: the presence of CS increases the chances of development of FM by more than 14 times (OR = 14.95; 95% CI 4.1744–53.5418, *p* < 0.01)” (results) “Our results showed a high prevalence of CS in patients with axSpA and a significant influence on QoL. The presence of CS significantly increases the potential risk of FM.” (Conclusion)
2021, Neurogastroenterology and Motility [8]	Department of Molecular and Clinical Medicine, Pain Rehabilitation, Centre for Functional GI and Motility Disorders	Sweden	“In this study, the presence and level of central sensitization, and its association to gastrointestinal (GI) symptoms were explored in…” (background) “The Central Sensitization Inventory (CSI) was translated and validated in Swedish and used together with the Highly Sensitive Person (HSP) scale to measure the presence and level of central sensitization.” (methods) “Central sensitization, defined by validated cut-off levels for CSI and HSP, was common in the whole cohort (40% and 28%) and in IBS (57% and 35%). Study participants with central sensitization had more severe GI symptoms, anxiety and depression, than participants without central sensitization.” (key results) “Central sensitization was common in IBS and associated with GI symptom severity, but with stronger associations in chronic pain disorders and IBD.” (conclusions and inferences)

3. Usefulness of the CSI for CS research: The CSI questionnaire may have good internal and external validity for identifying fibromyalgia symptomatology as a phenomenon [100,101,102,103], but that is not the same as measuring CS. Studies assessing the correlation between CSI scores and biomarkers supposedly indicative of CS [101] (e.g., GABA, BDNF) found only moderate correlation [7,20,104,105] and weak explanatory power. Cliton and colleagues (2021) evaluated the validity and the diagnostic accuracy of the CSI for detecting impairment in pain modulation in patients with chronic musculoskeletal pain [106]. They conclude that CSI is, in their words, a “useless instrument” for the detection of impairment in conditioned pain modulation in patients with chronic musculoskeletal pain. Goudman et al. (2023) found only a small correlation between pressure pain sensitivity and the CSI score in their study [2]. Furthermore, Adams et al. (2023) [107] conducted a systematic review with meta-analysis and examined whether the CSI is linked to enhanced nociceptive responses, as part of the supposed neurophysiological abnormalities of CS. According to their findings, the CSI strongly correlates with psychological constructs such as anxiety, stress, depression, pain catastrophizing, sleep, and kinesiophobia. The CSI showed weak or no correlations with experimental measures of nociceptive sensitivity such as pain thresholds, conditioned pain modulation, or temporal summation. They conclude that the CSI more closely reflects a psychological state than increased responsiveness of nociceptive neurons [107]. “The strong relationship between the CSI and thoughts and emotions suggests that the CSI functions largely as a mental health measure.” [108]. Similarly, Salbego et al. (2025) found that the CSI does not correlate with psychophysical parameters indicative of pain amplification in patients with temporomandibular disorders and is more associated with psychological or psychosocial factors (showing correlations with the Perceived Stress Scale, Pain Catastrophizing Scale, Pittsburgh Sleep Quality Index, and Hospital Anxiety and Depression Scale) (*p* < 0.001) and that 68.9% of the variation in CSI scores can be influenced all these variables (anxiety, depression, catastrophizing, and sleep) except stress [109]. (Anomalies are not a bad thing; they point in the right direction.) Neblett et al. (2024) [110] also conducted a systematic review and meta-analysis more recently to investigate the association between CSI scores and quantitative sensory testing (QST), which was published in a leading peer-reviewed journal in neuroscience. Their analysis compared groups of normal subjects, chronic pain subjects, and chronic musculoskeletal pain subjects, and the results were honestly fascinating. For conditioned pain modulation, out of all groups and in total, the best correlation found with the CSI score was r = −0.13. Temporal summation, pressure pain threshold, heat pain threshold, and cold pain threshold showed correlation coefficients of r = 0.16. r = −0.36, r = −0.17, and r = −0.27, respectively (these correlation values are the best out of all subject groups analyzed). That study is being cited by researchers in the field as supporting evidence for CSI as a valid tool for assessing CS [111].

The issue here is not whether central sensitization occurs or not, it is about proper science. Even if central sensitization occurs, it does not emerge from these studies. Researchers and clinicians should exercise caution when using CSI as a tool for CS without acknowledging these limitations. Authors conducting research on central sensitization should be aware that measuring an observed phenomenon (e.g., “fibromyalgia-type symptoms”, idiopathic pain, medical unexplained symptoms) while attributing it to a presupposed underlying mechanism—and then trying to measure that same mechanism by using the phenomenon as its proxy—is a methodologic tautology (Table 2 shows examples of this) and represents circular reasoning. CS is thus an axiom. The empirics are not the theory, and the theory should predict the empirics. Every postulated mechanism should explain and predict the phenomenon. The phenomenon is an outcome of its mechanism. To study the mechanism and its outcome, measurement instruments are needed. If the measurement tool is constructed from the phenomenon, it makes this tool an outcome of the mechanism. That is the first problem I wanted to address in my letter to you.

Borrowing words from TS Kuhn [112]: *“A person who builds an instrument to determine optical wave lengths must not be satisfied with a piece of equipment that merely attributes particular numbers to particular spectral lines. That person is not just an explorer or a measurer. On the contrary, that person must show, by analyzing this apparatus in terms of the established body of optical theory, that the numbers such an instrument produces are the ones that enter theory as wavelengths…”*.

## 3. Problem #2: Reliability and Validation of the CSI and Other Tools

The second problem pertains to reliability and validation. Irrespective of how it was constructed, the CSI tool has not been validated to measure hyperexcitability of nociceptive neurons or sensory augmentation in the central nervous system or spinal cord dorsal horn, and it is not derived from neuroscience’s theory, although it is regarded by clinical investigators and expert practitioners as a measure of CS. That is the second problem that is perpetuated in the field. Before using a tool, a scientist should ask the following: where did it come from, how was it constructed, was it validated, what is it validated for, and what does it measure? Placing your hand on a hot pan and then rating pain severity on a scale from 1 to 10 is not measuring temperature—does the reader agree? The Wells score, unfortunately, does not measure pulmonary embolus size. What do you think the Centor criteria measure?

Central sensitization is a neurophysiological alteration, and as such, it should be assessed by neurophysiological methods, not questionnaires. The phenomenon does not show you the mechanism. Fever, melancholy, and sore throat (i.e., “streptococcal pharyngitis symptoms”) are not valid scientific evidence of streptococcal infection because it can be viral. The appropriate way to use the CSI tool is not in circular reasoning but to correlate its score against an objective measure obtained from a neurophysiological method.

Even measures frequently proposed as indicators of central pain modulation do not represent reliable definitive biomarkers of central sensitization [43]. Nevertheless, the CSI is used in studies and still perceived as a good surrogate of an objective measure of CS, because there is no better tool for measuring/diagnosing CS in patients. With all the abovementioned disadvantages, CSI might be useful for clinicians to “diagnose” a syndrome/clinical picture, not a neurophysiological mechanism.

Future studies should aim to refine or develop reliable measurement tools that are theoretically grounded and capable of elucidating the underlying mechanisms of central sensitization.

## 4. Problem #3: Conceptual Ambiguity Regarding Central Sensitization

There is inconsistency across the literature on central sensitization. Some studies define central sensitization as a phenomenon [15,41,42,43,113,114,115,116,117] while others define it as a mechanism [14,31,40,61,115,116,118,119,120,121,122,123,124,125]. Some even discuss it as both a phenomenon and a mechanism in the same paper [115,126]. Table 3 shows examples of the conceptual ambiguity in the literature regarding central sensitization and the nociplastic pain paradigm across the field. If the phenomenon and the mechanism are conflated, that is a problem. If the phenomenon is also its own mechanism, the mechanism turns into an axiom.

Conceptual ambiguity regarding which is the phenomenon [15,41,42,113,114,115,116,117] and which is the mechanism [14,31,40,61,115,116,118,119,120,121,122,123,124,125] is the 3rd problem: “CS is an abnormal increased responsiveness of nociceptive neurons in the central nervous system to noxious and non-noxious stimuli, that is caused by a complex neuropathological process of…” [38,40,44,113,117,130,139,140,141,142] and, meanwhile, “CS is a complex neurophysiological process that occurs in the central nervous system that causes pain amplification and hypersensitivity to…” [84,101,122,124,126,140,142,143,144,145,146]. Pain researchers are gripping the rope at both heads, i.e., trying to have it both ways: it pulls on itself. Defining a theory by its outcome is leading to confusion in the field. “Nociplastic pain is a mechanistic term…” [61] and “key symptoms of nociplastic pain include pain in multiple body regions, fatigue, sleep disturbances, cognitive dysfunction, depression and anxiety…” [61,147]. There is a theory, and there are empirics. The empirics are not the theory. Is central sensitization a phenomenon or a mechanism? Is neuroplastic pain a phenomenon or a mechanism? Which is the phenomenon, and which is the mechanism being studied here? If central sensitization is your phenomenon [15,41,42,113,114,115,116,117], what is fibromyalgia? [148,149].

This ambiguity can lead to erroneous conclusions, and it complicates the synthesis of findings across studies, as researchers and/or readers may unknowingly refer to different constructs using the same terminology. To address these issues, a few recommendations are suggested: (i) authors should clearly define whether CS is being examined as a phenomenon or a mechanism in their studies. Academic medical journals can support this by encouraging explicit definitions and distinctions in submitted manuscripts. (ii) Theoretical framework: researchers should situate their studies within a theoretical framework that distinguishes between the phenomenon and the mechanism in their research study. There is a difference between studying pain and studying fibromyalgia. This will provide a clearer basis for drawing hypotheses and analyses.

“Central sensitization syndromes” seem to be the only medical condition whose name authors insist should be derived from a mechanism that has yet to be convincingly substantiated in research. Brazenor et al. (2022) [150], in a comprehensive literature review, found no convincing evidence showing that central sensitization can persist as an autonomous pain generator after initiating injury has healed. Moreover, Velasco et al. (2024) [151], in their recent review, note that they could not find a single study actually demonstrating central sensitization in humans (as opposed to hyperalgesia and other neurophysiological abnormalities, which have been shown in plenty of FM research studies). “Central pain augmentation” and “hyperalgesia” are, of course, not interchangeable terms. The former is a pillar of the theory, while the latter is part of the phenomenon (i.e., the syndrome of “fibromyalgia”). It is worth noting that “central sensitization”, as a phenomenon, is a term originally used in studies in animal models in the 1990s, was then borrowed in theory to try to help explain FM, and its true relevance and importance in the puzzle of FM syndrome is still under dispute and must be better understood through further empirical research. One has to admit that altered conditioned pain modulation and allodynia are not necessarily exclusive only to central sensitization.

One response is that some believe that CS research is in evolution, and it is important that in this process of evolution the terminology would be clearer and not cause disarray. A theory does not falsify or adjust the empirics. The empirics help falsify or adjust the theory. The term “human-assumed central sensitization” unjustifiably takes ownership of the phenomenon, and it does not leave room for alternative mechanisms to be considered.

“Fibromyalgia-type syndromes” is the phenomenon clinicians see in clinic, not CS. If the phenomenon being investigated is CS or “nociplastic pain”, the measurement instrument should be validated to measure hyperexcitability of nociceptive neurons in the central nervous system or spinal cord dorsal horn. If no available tool can measure it, pain research should work on constructing an instrument that can measure it. Physicists faced difficult challenges in the past and came up with the telescope.

## 5. Problem #4: Clinical Reasoning Is Being Conflated with Scientific Thinking

Clinical reasoning and scientific thinking are different. In clinical practice, for instance, the words diabetes mellitus II, hyperglycemia, and insulin resistance can be used somewhat interchangeably throughout a sentence, once the diagnosis has been made, because we know which medical entity it is. The symptoms are recognized, the manifestations are found, the underlying process is measured, and the mechanism is known to a degree sufficient for practical purposes. Polyuria and glucosuria are complementary to one another by means of osmotic pressure: the former is a symptom, and the latter is a finding. The empirics, in the main, correspond to what the contemporary scientific community thinks it knows about diabetes. As far as the clinician is concerned, the phenomenon and its mechanism are integrated into that medical entity. Likewise, new-onset chest pain with increasing levels of plasma cardiac troponin and ST segment alterations on electrocardiogram, following severe hypotension in a previously healthy young adult, represents a type II myocardial infarction. Catheterization is not necessarily indicated for it, and coronary bypass surgery probably will not help. This sort of clinical reasoning is problematic in the case of fibromyalgia-type syndromes. Here, the mechanism has not been proven, and the theory (theories) is (are) under dispute [45,46,47]. The phenomenon a clinician observes is fibromyalgia, not “human-assumed central sensitization”, [149] nor “primary pain,” [116,152,153,154], and not even “centralization of pain due to nociplastic central pain augmentation”. In this situation, the diagnosis of the syndrome is detached from the theory. The phenomenon is not the mechanism. It is the outcome of the mechanism. In science, we study a mechanism under a theoretical framework that should be adjusted according to empirics while using measurement tools that have been validated to measure the phenomenon (i.e., the outcome of the mechanism). Assessing fatigue and frequency of nocturia is not the same as a glucose tolerance test, using diabetes as an analogy. The field, instead of assuming CS and fixating on semantics, should start focusing efforts on the development of valid measurement tools, theory-driven biomarkers, multimodal assessment frameworks, longitudinal validation studies, and improved conceptual definitions of nociplastic pain and central sensitization.

There are many theories for fibromyalgia [45,46,47,53,55,152,155,156,157,158,159,160,161,162,163,164,165,166,167,168,169,170], and a researcher is free to choose which makes the most sense.

## 6. Problem #5: Terminology Issues

Another problem to note is that some of the literature on fibromyalgia has the term “central sensitivity syndromes” [171,172] (also “central sensitization syndromes”) [173,174], which is used by researchers for referring to fibromyalgia-type syndromes. What happens is that naming the phenomenon by the name of a mechanism inherently made a presumed connection between the phenomenon and the theory. Thus, the assumption is made by the listener that the mechanism of this phenomenon is, obviously, central sensitization. It is spelled out rather clearly. Autoimmune thyroiditis is an autoimmune condition, without doubt, even the textbooks describe it as such. What is the mechanism of a “central sensitivity syndrome”? Disseminated intravascular coagulation is an acute life-threatening activation of coagulation within the vasculature that is diffuse (or disseminated), and inflammatory bowel disease is a disease characterized by inflammation of the bowel—it is self-explanatory, consistent with common understanding, and supported by the literature. Terminology ought to respect the observed natural phenomenon. Fibromyalgia-type syndromes are the observed phenomenon. In sickle cell disease, you can find sickled erythrocytes. What do we find in central sensitization syndromes? Go back to the story of the tower of Babylon (Genesis 11). Nomenclature is part of human language whereas the phenomenon is a reality of nature. The primary fallacy resulted not from flawed terminology, but from flawed thinking.

“This tool is a good measure”: a measure of what???

It seems that, in the field of pain research, there is no distinction made between the measurement instrument, the phenomenon, and the theory. This might be part of the reason for why there are so many anomalies.

## Figures and Tables

**Table 3:** Examples of central sensitization as both a phenomenon and a mechanism. There is a phenomenon. The phenomenon has a mechanism. The phenomenon does not show you the mechanism. The phenomenon is the outcome of the mechanism.

References on Central Sensitization/Nociplastic Pain as a Phenomenon	References on Central Sensitization/Nociplastic Pain as a Mechanism
“Central Sensitization (CS) is a proposed physiological phenomenon in which central nervous system neurons become hyper-excitable…” [15]	“Central sensitization is considered a key mechanism…” [118]
“…the phenomenon of central sensitization.” [113]	“…and central mechanisms (e.g., central sensitisation)” [31]
“It is known that fibromyalgia is caused by a central sensitization phenomenon…” [115]	“Central sensitization refers to a neuronal signal amplification mechanism within the central nervous system that leads to a greater perception of pain. For this reason, patients with FM present an increase in the receptive field of pain…” [115]
“Possible Mechanisms Causing Central Sensitization…” [117]	“Central Sensitization (CS) has been proposed as a common pathophysiological mechanism to explain related syndromes for which no specific organic cause can be found” [14]
“It seems important, therefore, to distinguish “central sensitization” which is a neurophysiological phenomenon…” [116]	“The relevant pain mechanisms (nociceptive, neuropathic and/or nociplastic pain)…” [127]
“As in other chronic pain conditions, this might be the pathogenic mechanism primarily responsible for maintaining pain—a phenomenon now referred to as nociplastic pain.” [128]	“The reason is that sensitization, a key pathophysiological mechanism in nociplastic pain…” [40]
“…the construct of “nociplastic pain” describes a phenomenon that…” [116]	“It is important to keep in mind that ‘nociplastic’ is a mechanistic term, ‘chronic primary pain’ a diagnostic term (disease classification) and ‘central sensitization’ a neurophysiological term” [127]
“When considered together, the findings discussed above suggest that nociplastic pain is ultimately a CNS phenomenon” [61]	“Mechanistic terms such as “nociplastic-“, “neuropathic-“, or “nociceptive pain” describe mechanisms.” [129]
“At the spinal level, central sensitization encompasses active amplification of sensory stimulation, which enhances pain response to noxious stimuli, and recruitment of normally subliminal low-threshold sensory inputs, which can then activate the pain circuit. One such mechanism is temporal summation…” [130]	“Central sensitization: A neurophysiological process of pain amplification in the central nervous system.” [122]
“Another term that has often been used to describe a similar phenomenon is “central sensitization.”” [43] “Because both central pain and central sensitization have these historical meanings, we have typically used terms such as central augmentation or amplification to refer more broadly to the many CNS mechanisms that enhance the perception or modulation of pain differentially between individuals, and we have used the term “centralized pain” to indicate that this phenomenon plays a role in any individual with chronic pain.” [43]	“This term was originally coined to refer to a specific spinal cord mechanism that we now realize as being one of many potential causes of augmented CNS pain processing” [43] “…peripheral/nociceptive, peripheral neuropathic, or centralized, this is meant to indicate the primary underlying mechanism for pain in each of these diagnoses” [43]
“The origins of central sensitization in pain research can be traced to the seminal work of Clifford Woolf, who, in the 1980s, first described the phenomenon in basic science studies of spinal cord neuroplasticity and its role in chronic pain” [126]	“Central sensitization, which refers to the heightened responsiveness of the CNS to sensory stimuli, has been identified as a key mechanism not only in fibromyalgia…” [126]
“Nociplastic pain is defined as pain that arises from altered nociception with sensitisation as the major underlying mechanism” [31]	“The main pathophysiological mechanism for developing nociplastic pain is central sensitization (CS) in which pain amplification and hypersensitivity occur. Fibromyalgia is the prototypical nociplastic pain disorder…” [131]
“Chronic pain is maintained in part by central sensitization, a phenomenon of synaptic plasticity…” [132]	“Although the facilitated degree of temporal summation indicates an enhanced central integrative mechanism (i.e., central sensitization)” [133]
“…whereas pain is an emergent phenomenon…” [133]	“…Identify key clinical features and mechanisms of central pain syndrome, including central sensitization,” [134] “Central pain syndrome involves pathophysiological mechanisms distinct from those underlying nociplastic pain conditions, such as fibromyalgia.” [134]
“Thanks to the discovery of the phenomenon of central sensitization (CS)…” [135]	“A further consideration concerns the homogeneity of categorizations such as Nociceptive pain (NP), peripheral neuropathic pain (PNP), and central sensitization pain (CSP), that is the extent to which patients classified with a given dominant pain state have pain attributable to common pathophysiological mechanisms.” [136]
“As the use of the term central sensitization has expanded over the past several decades, so has our understanding of CNS processes that may lead to this phenomenon.” [137]	“Multiple mechanisms that can produce pain have been identified; they include nociception, peripheral sensitization, phenotypic switches, central sensitization…” [125]
“Enhanced temporal summation has been detected in multiple pain conditions, and is considered a key mechanism of central sensitization.” [138]	“The mechanisms that underlie this type of pain are not entirely understood, but it is thought that augmented CNS pain and sensory processing and altered pain modulation play prominent roles.” [84]

## Data Availability

Data are contained within the article.

## Abbreviations

The following abbreviations are used in this manuscript:

CS	central sensitization
CSI	central sensitization inventory
FM	fibromyalgia
